# A novel function of RHOA as a host-dependent factor in *Glaesserella parasuis* infection of LLC-PK1 cells

**DOI:** 10.1186/s13567-025-01617-6

**Published:** 2025-09-25

**Authors:** Huanhuan Zhou, Xuexue Chen, Xinqi Zeng, Shengsong Xie, Xiaoyu Zhang, Jiayi Zeng, Ke Xu, Bo Yu, Hailong Liu, Hongbo Chen

**Affiliations:** 1 Laboratory of Genetic Breeding, Reproduction and Precision Livestock Farming, School of Animal Science and Nutritional Engineering, Wuhan Polytechnic University, Wuhan, 430023 China; 2 Hubei Provincial Center of Technology Innovation for Domestic Animal Breeding, Wuhan Polytechnic University, Wuhan, 430023 China; 3 Key Laboratory of Agricultural Animal Genetics, Breeding and Reproduction of Ministry of Education & Key Lab of Swine Genetics and Breeding of Ministry of Agriculture and Rural Affairs, Huazhong Agricultural University, Wuhan, 430070 China

**Keywords:** *Glaesserella parasuis*, pig, *RHOA*, adhesion, invasion

## Abstract

*Glaesserella parasuis* (*G. parasuis*), a lethal pathogen causing Glässer’s disease, poses severe threats to global swine health. While the small GTPase Ras homolog gene family member A (*RHOA*) is implicated in viral pathogenesis, its role in bacterial infections remains unexplored. Here, we established an in vitro infection model using porcine LLC-PK1 cells and demonstrated that *G. parasuis* induces adhesion and pseudopodia-mediated invasion, resulting in approximately 99% cell death within 120 h post-infection. Crucially, *RHOA* expression was upregulated during infection, and *RHOA* knockout reduced bacterial adhesion and invasion, rescuing cell viability to 77.30%. Transcriptomic profiling of *RHOA*-knockout cells revealed 1797 differentially expressed genes, revealing indirect effects on cytoskeleton remodeling (*ACTG1*/*MYL7/MYL9* downregulation) and tight junction stabilization (*CDH1*/*CLDN1/CDH5* upregulation). This establishes *RHOA* as a key host factor facilitating *G. parasuis* infection, providing targets for disease control.

**Supplementary Information:**

The online version contains supplementary material available at 10.1186/s13567-025-01617-6.

## Introduction

*Glaesserella parasuis* (*G. parasuis*) is a short rod-shaped gram-negative bacterium that is recognized as an opportunistic pathogen that initially colonizes the upper respiratory tract of healthy pigs [1, 2]. Under certain conditions of stress, it can breach the mucosal barrier and enter the bloodstream, promoting a systemic inflammatory response. Following *G. parasuis* infection, pigs present with Glässer’s disease, which is characterized by multifocal serositis, pneumonia, arthritis, and meningitis [3]. At least 15 serovars of *G. parasuis* have been reported, with the predominant serotypes in China ranked by virulence (from strong to weak) as serotypes 5, 13, 4, and 12 [4]. These serotypes can directly cause host mortality and are often associated with co-infections with other bacteria or viruses [5]. The complexity of *G. parasuis* infection, combined with our limited understanding of host interactions, hinders the development of effective vaccines and antibacterial strategies for Glässer’s disease.

During the colonization of the upper respiratory mucosa, *G. parasuis* expresses immunoglobulin A (IgA) proteases that degrade the heavy chain structure of porcine IgA, allowing the bacteria to evade the host's mucosal defenses [6]. This facilitates the breach of the respiratory epithelial barrier [7, 8], creating circumstances conducive for bacterial entry into the bloodstream and subsequent dissemination to the lower respiratory tract [9]. In the lung, highly virulent strains can evade phagocytosis by porcine alveolar macrophages [10, 11] and resist bactericidal effects from serum components [12], triggering a strong cellular inflammatory response. These evasion mechanisms further facilitate bacterial spread through the bloodstream and systemic dissemination. Furthermore, *G. parasuis* can adhere to and invade various epithelial/endothelial cells at multiple anatomical sites, including the lungs, blood vessels, kidneys, brain, and other organs [3], thus establishing a general pathway for bacterial infection from the primary invasion site to secondary replication or pathogenic foci throughout the body.

Recent studies have indicated that *G. parasuis* spreads by disrupting the cell‒cell adhesions (also known as intercellular junctions) of host epithelial [7, 8, 13, 14] and endothelial barriers [15]. Normal intercellular junctions rely on the localized regulation of the actin cytoskeleton by the Rho GTPase family [16]. RhoA (encoded by *RHOA*), a key regulator of the cytoskeletal system, modulates various actin-driven processes and mediates multiple viral infections [17, 18]. During early infection with porcine sapovirus (PSaV), activation of the RhoA/ROCK/MLC signaling pathway induces contraction of the actomyosin ring, compromising tight junction integrity and exposing the core receptor occludin to facilitate viral spread [19, 20]. Similarly, classical swine fever virus (CSFV) requires the activation of RhoA-related pathways in host cells to regulate F-actin dynamics, aiding in CSFV endocytosis [21]. Conversely, RhoA acts as a host restriction factor for porcine pseudorabies virus (PRV). Its overexpression or activation can induce actin cytoskeleton remodeling and inhibit PRV replication [22]. These findings indicate that the same gene can perform dual functions in response to different pathogens.

Despite its significance in viral infections, *RHOA*'s function in bacterial diseases like *G. parasuis* remains unknown. As a GTPase signaling node, *RHOA* indirectly influences transcription through effectors (e.g., ROCK [23]) and host transcription factors (e.g., NF-κB [24]), altering gene expression [25]. For instance, *RHOA* controls SRF-dependent genes during smooth muscle cell differentiation via actin dynamics and Rho kinase [26]. This study investigates the pathogenic relationship between *RHOA* and *G. parasuis* using an in vitro infection model with LLC-PK1 cells, including transcriptomic analysis to define indirect mechanisms underlying bacterial pathogenesis. Our findings provide new insight for understanding *G. parasuis* infection and corresponding molecular breeding for disease resistance.

## Materials and methods

### Cells and bacterial strains culture

The *G. parasuis* SH0165 strain (serotype 5) was cultured at 37 °C in tryptic soy broth (TSB) (Difco, BD, USA) or on tryptic soy agar (TSA) (Difco, BD, USA). The media were supplemented with 10% fetal bovine serum (FBS) (Gibco, California, USA) and 10 µg/mL nicotinamide adenine dinucleotide (Beyotime, Shanghai, China). A single colony was selected and inoculated into TSB, where it was incubated overnight at 37 °C with shaking at 220 rpm until the optical density at 600 nm (OD600) reached 0.6–0.7. Porcine renal proximal tubule epithelial cells (LLC-PK1) were cultured in minimum essential medium (Gibco, California, USA) supplemented with 10% FBS (Cell-Box, Hong Kong, China) at 37 °C in a humidified cell incubator (Thermo Fisher Scientific, Massachusetts, USA) with 5% CO_2_.

### Cell viability assay

Cell viability was assessed via a CellTiter-Lumi luminescent assay kit (Beyotime, Shanghai, China) following the manufacturer’s instructions. The cells were digested with 0.25% EDTA trypsin (Gibco, California, USA) and transferred to a 96-well plate. An equal volume of CellTiter-Lumi reagent was added. After shaking for 2 min, the samples were incubated for 10 min at room temperature. Cell viability was then measured via an LB960 XS3 luminometer (Berthold, Baden-Württemberg, Germany). Each condition included three biological replicates and three technical replicates.

### Adhesion and invasion assays

The assays were carried out as previously reported [27]. The cells were seeded in 24-well cell plates and challenged with *G. parasuis*. To evaluate the number of adhered bacteria, the cells were washed five times with sterile PBS to prevent nonspecific attachment. The samples were treated with 200 µL of 0.25% trypsin–EDTA (Gibco, California, USA) at 37 °C for 10 min. After trypsinization, the cells were lysed with 800 µL of precooled deionized water and mixed vigorously to ensure complete lysis and bacterial release. For the invasion experiment, the cells underwent similar steps, but the extracellular bacteria were killed by incubation with culture medium containing 10 µg/mL penicillin G (Biosharp, Anhui, China) and 100 µg/mL gentamicin (Biosharp, Anhui, China) for 1 h. A 100 µL aliquot of the cell suspension was plated on TSA plates and incubated at 37 °C for 36 h for colony-forming unit (CFU) counting. Three biological replicates and three technical replicates were included for each condition.

### Scanning electron microscopy (SEM) analysis

The cells were washed three times with PBS and then fixed at room temperature for 2 h using an electron microscopy fixative containing 2.5% glutaraldehyde in 0.1 M phosphate buffer (Servicebio, Wuhan, China). After three additional washes, the cells were postfixed with 1% osmium tetroxide (Ted Pella, California, USA) at room temperature in the dark for 2 h. The samples were then dehydrated through a series of increasing concentrations of ethanol. The samples were subsequently dried via a critical point dryer (Quorum, UK) and coated with gold via an ion sputter coater (HITACHI, Tokyo, Japan). Observations and imaging were performed with a SU8100 scanning electron microscope (HITACHI, Tokyo, Japan).

### Transmission electron microscopy (TEM) analysis

The cells were gently washed with PBS and fixed in electron microscopy fixative at 4 °C for 2 h. Following fixation, the samples were rinsed with PBS and treated with osmium tetroxide (SPI-Chem, West Chester, USA). After additional washes, the samples were dehydrated via a series of ethanol and acetone solutions. The samples were then embedded, trimmed, and sectioned. The sections were collected on copper grids and stained with lead and uranium stains, followed by washing with ultrapure water and drying. Finally, the samples were examined via an HT7800 transmission electron microscope (HITACHI, Tokyo, Japan).

### Real-time quantitative PCR (RT‒qPCR)

Total RNA was extracted from cells via TRIzol reagent (Invitrogen, California, USA). After the quality of the RNA was assessed, cDNA was synthesized via the PrimeScript RT Reagent Kit with gDNA Eraser (Takara Bio, Shiga, Japan). RT‒qPCR was performed according to the instructions of TB Green Premix Ex Taq II (Takara Bio, Shiga, Japan) on a QuantStudio 1 Plus Real-Time PCR System (Thermo Fisher, Waltham, USA). Each well in the 96-well plate contained 5 μL of TB Green II, 3.6 μL of RNase-free water, 1 μL of cDNA, 0.2 μL of forward primer, and 0.2 μL of reverse primer, with GAPDH as the internal control. The reaction included initial denaturation at 95 °C for 30 s, followed by 40 cycles of amplification (95 °C for 5 s and an appropriate annealing temperature of 30 s). Each sample was run in triplicate, and the data were analyzed via the 2^−△△Ct^ method [28]. The primers used for this study are listed in Table 1.

**Table 1:** **Primers information for the mRNAs used for RT‒qPCR**

Gene	Sequences (5' → 3')	Size (bp)	Anneal temperature (°C)
*GAPDH*	F:TGACATCAAGAAGGTGGTGAAG	159	58
	R:TTGACGAAGTGGTCGTTGAG		
*RHOA*	F:CGTTGGTCTTGCAGCACATT	127	60
	R:TACCTCCGGGAATTGGTCCT		
*IFIT1*	F:AGAGGAGCCCATCCAGCTAAA	190	63
	R:TCAATCTCCTCCAAGACCCTG		
*MYL9*	F:TGCAGGAGTTCACACCCATC	117	61.4
	R:TGCAGGAGTTCACACCCATC		
*RSAD2*	F:TGTCCTTATTGGCCGTGGTC	295	63
	R:CACGTCTTTGTGGCGGTCTA		
*GREM1*	F:CTGTTCTCCAAGAAGCTGAGTCT	197	63
	R:CTGCTGGGGAGATTGAGTCTG		
*RGS4*	F:TCCTAACGTGAATCCCACACT	283	63
	R:ACTGTATTCAGACTTCAGGAAAGC		
*ABCA12*	F:AGTCCAGGTCCAAGCGATTC	191	60.8
	R:TGCAGGAGTTCACACCCATC		
*CDH1*	F:GACACCCGGGACAACGTTTA	234	58.6
	R:GGCAGTGGGGTCACTATCAG		
*CLDN1*	F:TACCCAACACCAAGGCCCTA	275	58.6
	R:ACACATGAAAATGGCTTCCCT		

### Generation of *RHOA* knockout (*RHOA*-KO) LLC-PK1 cells

*RHOA*-KO LLC-PK1 cells were generated via CRISPR/Cas9 technology and a lentiviral packaging system. The specific small guide RNA (sgRNA) sequence targeting porcine *RHOA*, 5’-GAGGTATATGTACCTACCGT-3’, was subsequently cloned and inserted into the Lenti-sgRNA-EGFP vector. For lentivirus production, HEK293T cells were transfected with the Lenti-*RHOA*-KO plasmid, psPAX2 (Addgene #12260), and pMD2. G (Addgene #12259) at a 3:2:1 ratio using jetPRIME transfection reagent (Polyplus, Strasbourg, France) following the manufacturer’s instructions. After 72 h, the supernatant was collected and filtered through a 0.45 μm filter (Biosharp, Anhui, China). The virus was concentrated via an Optima XE-90 ultracentrifuge (Beckman, California, USA) for 3 h at 4 °C. The purified lentivirus was then used to infect LLC-PK1 cells in the presence of polybrene transfection reagent (Millipore, Massachusetts, USA). The EGFP-positive cells were subsequently sorted via flow cytometry (BD, New Jersey, USA) to obtain *RHOA*-KO LLC-PK1 cells, which were subsequently verified via DNA sequencing and western blotting. The forward primer used for sequencing was 5’-GGCCTGAGACTATTATGGCTT-3’, and the reverse primer was 5’-CACATATTCTAAGGCTTCCCAATG-3’.

### Western blot

The cells were rinsed three times in ice-cold PBS and lysed in RIPA buffer (Beyotime, Shanghai, China) containing protease inhibitors on ice. The cell lysates were subsequently centrifuged at 12 000 × *g* for 15 min. Proteins in the supernatant were collected and quantified via a BCA protein assay kit (Beyotime, Shanghai, China). The proteins were then boiled with 5 × SDS‒PAGE sample loading buffer (ABclonal, Wuhan, China) for 10 min, separated via SDS‒PAGE (Bio-Rad, Hercules, USA), and transferred to a 0.22 μm PVDF membrane (Millipore, Massachusetts, USA). The membrane was blocked with 5% skim milk for 2 h at room temperature and incubated overnight with specific primary antibodies at 4 °C. After the primary antibody incubation, the membrane was incubated with HRP-goat anti-mouse IgG H + L (1:8000, Proteintech, #SA00001-1, China) or HRP-goat anti-rabbit IgG H + L (1:5000, ABclonal, #AS014, China) for 1 h at room temperature in the dark. The protein bands were visualized via an enhanced chemiluminescence (ECL) detection kit (Beyotime, Shanghai, China) and a QuickChemi 5200 imaging system (v1.1.5.0, Monad, USA). The primary antibodies used were anti-RHOA rabbit pAb (24 kDa, 1:800, Wanleibio, #WL02853, China) and anti-β-actin mouse mAb (42 kDa, 1:80 000, Proteintech, #66009-1, China).

### EdU proliferation assay

Cell proliferation was assessed via the BeyoClick^™^ EdU Cell Proliferation Kit with Alexa Fluor 555 (Beyotime, Shanghai, China) according to the manufacturer’s instructions. The cells cultured in six-well plates were incubated with 10 μM EdU working solution for 2 h. The cells were subsequently fixed with 4% paraformaldehyde (Solarbio, Beijing, China) at room temperature for 15 min. After three washes, the cells were incubated with immunostaining permeabilization buffer (Beyotime, Shanghai, China) at room temperature for 15 min, followed by two washes. The cells were then incubated with Click reaction mixture at room temperature in the dark for 30 min. After being washed, the cells were stained with Hoechst 33342 at room temperature in the dark for 10 min. Following three washes, fluorescence detection was performed under an inverted fluorescence microscope (Life Technologies, California, USA). The quantification of EdU-positive cells was carried out via ImageJ software (v1.46r).

### Giemsa staining

The cells in the six-well plates were washed three times with PBS at 37 °C, followed by fixation with 70% ethanol for 10 min. The samples were then stained with 1 × Giemsa staining solution (Beyotime, Shanghai, China) at room temperature for 45 min. After staining, the samples were carefully washed with PBS and air-dried. Images were captured via an optical microscope (MSHOT, Guangzhou, China).

### mRNA library preparation

Total RNA was isolated via TRIzol Reagent (Takara Bio, Shiga, Japan). The quantity and integrity of the extracted RNA were assessed via a Qubit 4.0, Agilent 2100, and NanoDrop ND-2000 spectrophotometer. mRNA was enriched from total RNA via the HieffNGS^®^ mRNA Isolation Master Kit (Yeasen, Shanghai, China) with oligo(dT) magnetic beads. Next, the mRNA was fragmented and converted into double-stranded cDNA. Following this, end repair and poly(A) tailing were performed. After the sequencing adapters were ligated, purification and size selection were carried out via HieffNGS^®^ DNA Selection Beads (Yeasen, Shanghai, China). Finally, PCR amplification was conducted to generate the library. Once the library passed quality control, high-throughput sequencing was performed via a DNBSEQ-T7 sequencer (BGI Tech, Shenzhen, China).

### Transcriptomic analysis

The raw reads were filtered via fastp (v0.21.0) to obtain clean reads. The sequences were then aligned to the Sscrofa11.1 reference genome via HISAT2 (v2.1.0). Gene expression analysis was conducted via StringTie (v2.1.5), with expression levels normalized to fragments per kilobase of transcript per million fragments mapped (FPKM) values. Differential expression analysis was performed via DESeq2 (v1.30.1) with the criteria |FoldChange|≥ 2 and padj ≤ 0.05. Gene Ontology (GO) and Kyoto Encyclopedia of Genes and Genomes (KEGG) enrichment analyses were carried out via clusterProfiler software, and the hypergeometric distribution method was used to calculate p values (with a significance threshold of padj < 0.05). The results were visualized via the OmicShare Tool [29]. Pathway maps were visualized using Cytoscape (v3.10.3), based on KEGG-enriched differentially expressed mRNAs.

### Statistical analysis

All experiments included a minimum of three biological replications. The data are presented as the means ± standard deviations (SDs) and were analyzed via GraphPad Prism 8 (v8.3.0.538) via two-tailed t-tests. Adobe Illustrator 2021 (v25.0.1) was used for plotting. A value of *p* < 0.05 was considered statistically significant.

## Results

### Upregulation of *RHOA* in LLC-PK1 cells infected with *G. parasuis*

**Figure 1:**
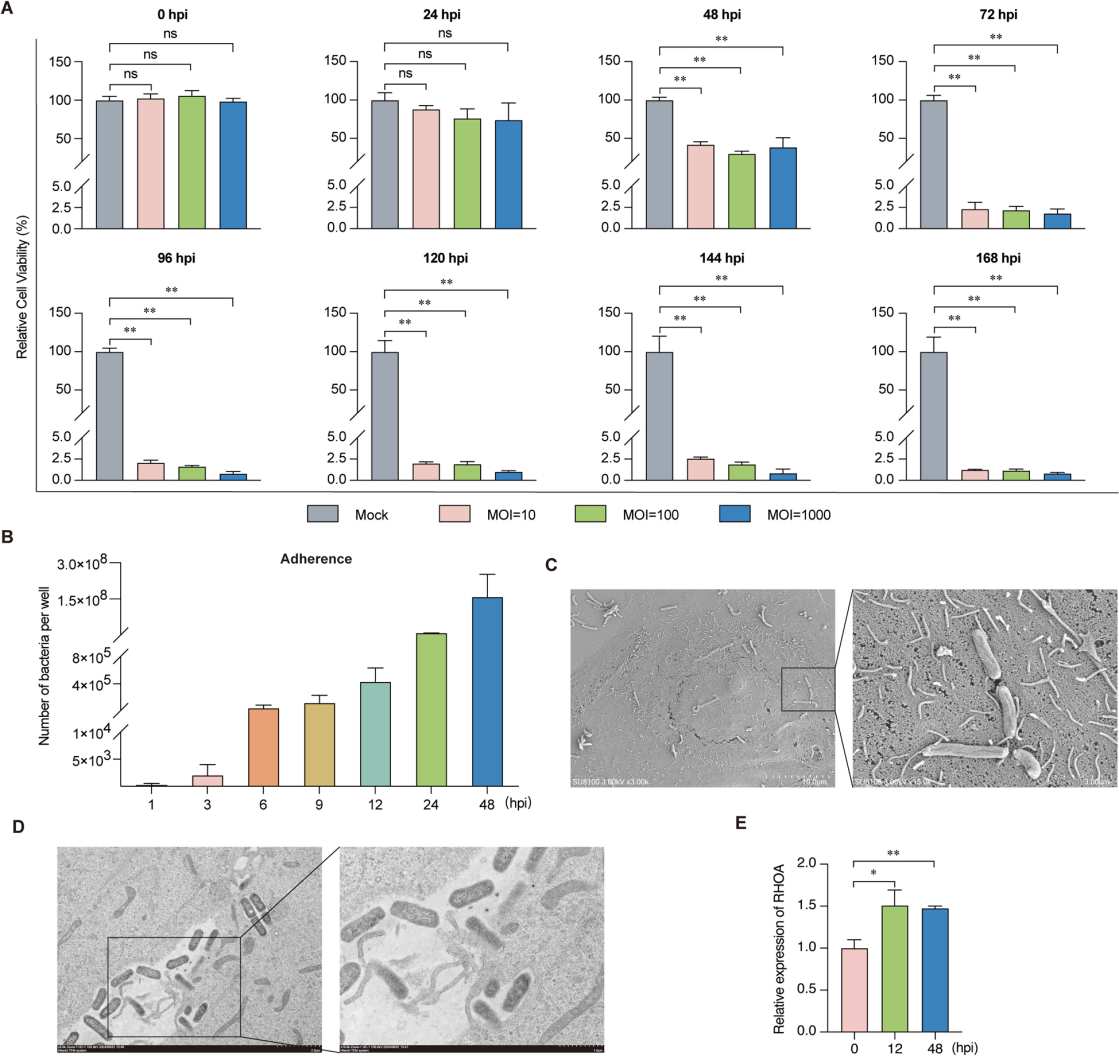
***RHOA* expression in *G. parasuis*-infected LLC-PK1 cells. A** Cell viability of LLC-PK1 cells infected with *G. parasuis* at MOIs of 10, 100, and 1000.** B** Quantification of adhered bacteria at the indicated time points. **C** SEM image of bacterial adhesion at 24 hpi (MOI = 10). **D** TEM image of bacterial invasion (48 hpi, MOI = 10).** E** RT‒qPCR analysis of *RHOA* mRNA levels (MOI = 10). The sample size is “*n* = 3”. ns indicates no significant difference; ^*^p < 0.05; ^**^p < 0.01.

To investigate the role of the porcine *RHOA* gene during *G. parasuis* infection, we established an in vitro lethal infection model using LLC-PK1 cells challenged with *G. parasuis* serotype 5. The viability of cells exposed to *G. parasuis* at multiplicities of infection (MOI) of 10, 100, and 1000 decreased in a time-dependent manner. Compared with that of mock-treated cells, cell viability decreased to approximately 38% at 48 hpi, followed by a rapid decrease to approximately 2% at 72 hpi and nearly complete cell death (approximately 1% viability) at 168 hpi (Figure 1A). Given the similar cell viability trends across MOIs, subsequent experiments focused on an MOI of 10.

Quantification of bacterial adhesion revealed a progressive increase in *G. parasuis* adhesion to LLC-PK1 cells over time (Figure 1B). Scanning electron microscopy (SEM) confirmed bacterial adhesion to the cell surface (Figure 1C), whereas transmission electron microscopy (TEM) demonstrated the engulfment and internalization of *G. parasuis* associated with pseudopodia formation (Figure 1D). Notably, *RHOA* mRNA expression was significantly upregulated at 12 hpi and 48 hpi compared with that at 0 hpi (Figure 1E), which correlated temporally with bacterial adhesion dynamics.

### Knockout of *RHOA* enhances host cell survival during lethal* G. parasuis* infection

**Figure 2:**
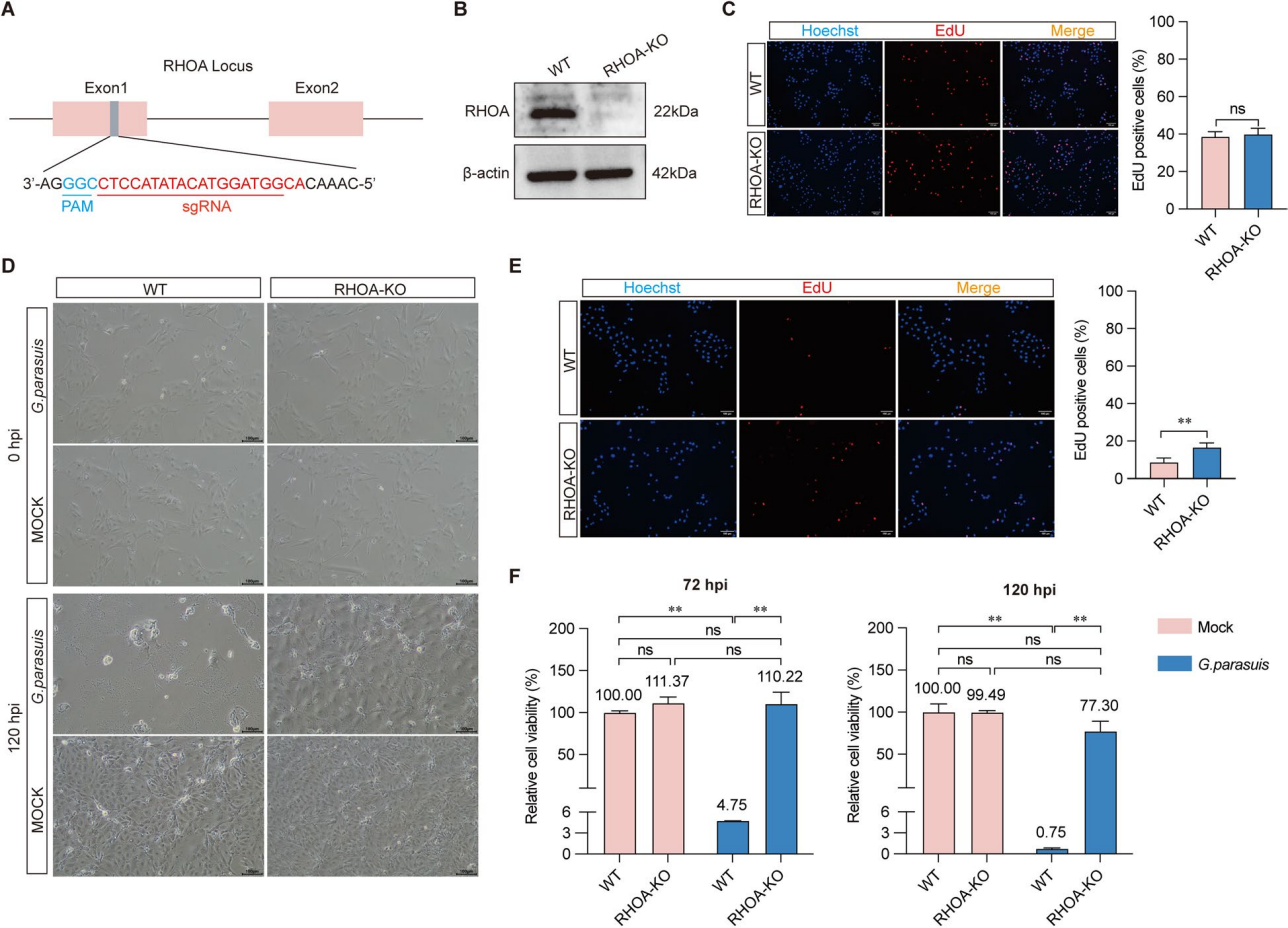
***RHOA* knockout mitigates *G. parasuis*-induced cytopathic effects. A** Schematic of sgRNA targeting *RHOA*. **B** Western blot confirming *RHOA* knockout. **C** EdU assay showing cell proliferation in *RHOA*-KO vs. WT cells. **D** Morphological comparison of cells at 0 and 120 hpi. Magnification: 100 × ; scale bar: 100 μm. **E** Quantification of EdU-positive cells in the WT and *RHOA*-KO groups at 48 hpi. Magnification: 100 × ; scale bar: 100 μm. **F** Cell viability at 72 hpi and 120 hpi. The sample size is “*n* = 6”. ns, not significant; ^*^p < 0.05; ^**^p < 0.01.

*RHOA*-knockout (*RHOA*-KO) LLC-PK1 cells were generated via CRISPR/Cas9, and successful knockout was confirmed via western blotting (Figures 2A, B). EdU staining confirmed that *RHOA* depletion did not alter baseline cell proliferation (Figure 2C). Following *G. parasuis* infection (MOI = 10), wild-type (WT) cells exhibited severe cytopathic effects (swelling, rupture, and detachment) by 120 hpi, whereas *RHOA*-KO cells retained normal morphology and viability (Figure 2D). The quantification of the EdU-positive cells revealed significantly greater proliferation in the *RHOA*-KO cells than in the WT cells post-infection (Figure 2E). Cell viability assays revealed a dramatic 103-fold increase in survival (from 0.75% in WT to 77.30% in *RHOA*-KO cells) at 120 hpi (Figure 2F).

### Depletion of *RHOA* reduces *G. parasuis* adhesion and invasion

**Figure 3:**
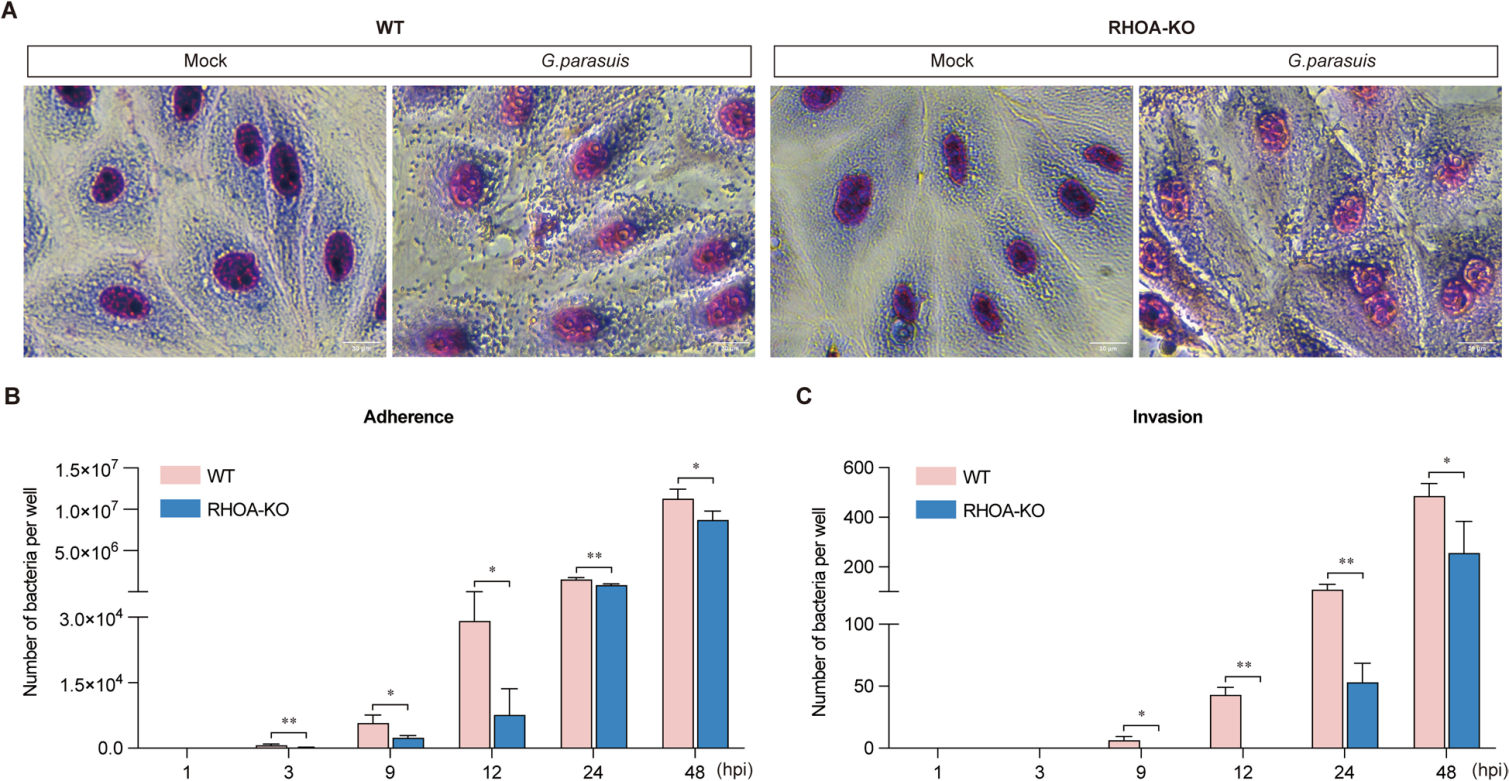
**Assessment of *G. parasuis* adhesion and invasion at different incubation time points (MOI = 10). A** Giemsa staining of adhered bacteria (24 hpi). Magnification: 400 × ; scale bar: 30 μm. **B** Quantification of bacterial adhesion. **C** Quantification of bacterial invasion. The sample size is “*n* = 3”. ns, not significant; ^*^p < 0.05; ^**^p < 0.01.

Giemsa staining revealed fewer adherent bacteria on *RHOA*-KO cells than on WT cells at 24 hpi (Figure 3A). The quantification of colony-forming units (CFUs) revealed significant reductions in bacterial adhesion (3 hpi, 9 hpi, 12 hpi, 24 hpi, and 48 hpi) and invasion (9 hpi, 12 hpi, 24 hpi, and 48 hpi) in *RHOA*-KO cells (Figures 3B, C). These results indicate that *RHOA* depletion impairs *G. parasuis* adhesion and invasion, effectively mitigating infection.

### Identification of differentially expressed (DE) mRNAs

**Figure 4:**
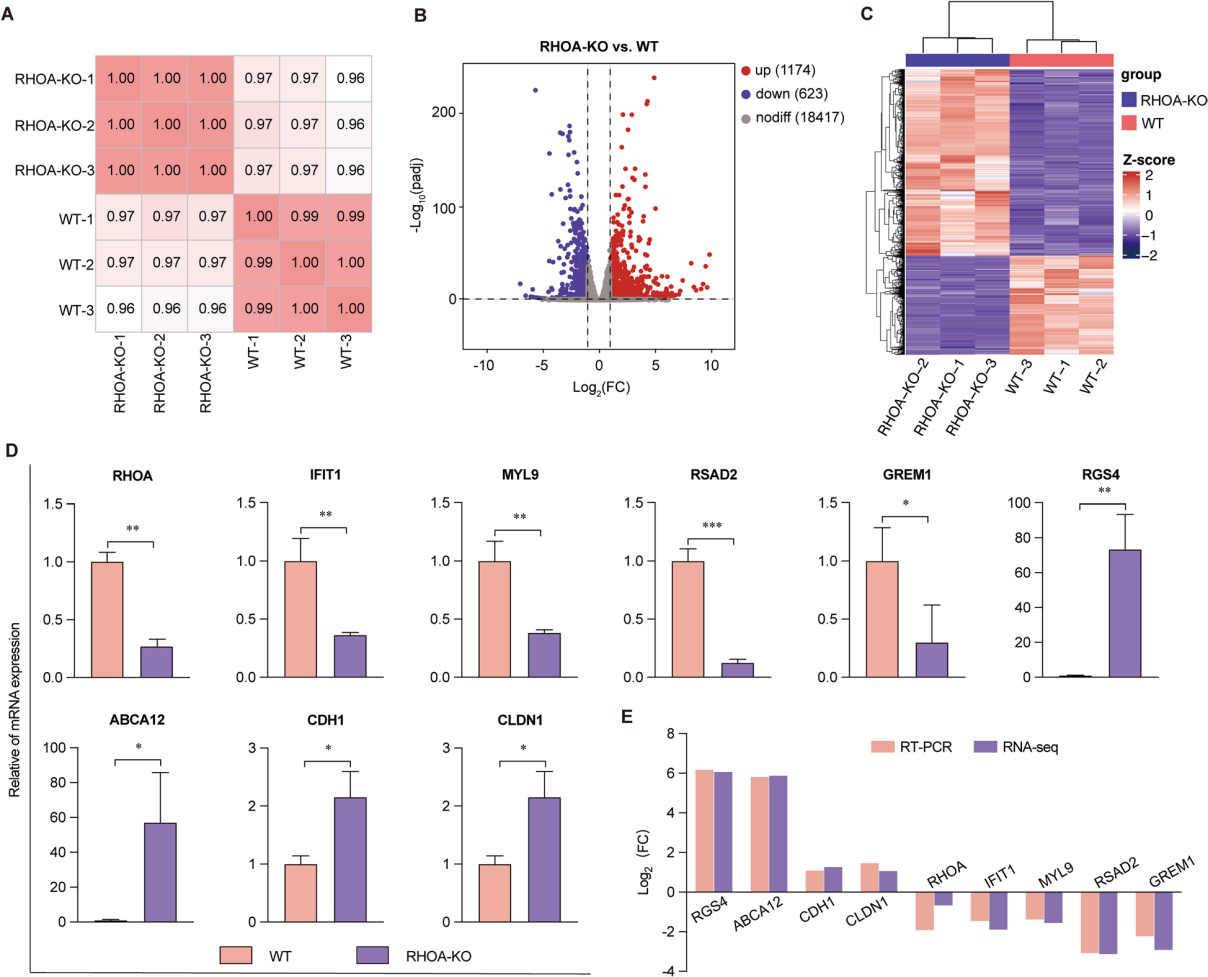
**Transcriptomic profiling of RHOA-KO cells. A** Correlation heatmap of biological replicates.** B** Volcano plot of DE genes. **C** Heatmap of DE gene expression profiles. **D** RT‒qPCR validation of selected DE mRNAs. **E** Comparison of mRNA expression patterns obtained from RT‒qPCR and RNA‒seq methodologies, presented as log_2_(FC). ^*^p < 0.05; ^**^p < 0.01.

RNA sequencing of WT and *RHOA*-KO cells generated high-quality data (Q30 > 96.76%; alignment rate: 89.36–94.34%) (Table 2). Correlation analysis among biological replicates revealed a correlation coefficient over 0.99 (Figure 4A). Differential expression analysis revealed a total of 1,174 upregulated and 623 downregulated genes following *RHOA* knockout (Figure 4B; Additional file 2). Additionally, bi-clustering analysis revealed that the expression profiles of these 1,797 DE mRNAs clustered as anticipated between the WT and *RHOA*-KO groups, with similar expression levels within groups and divergent levels between groups (Figure 4C). To validate the RNA-seq data, four downregulated genes (*IFIT1*, *MYL9*, *RSAD2*, and *GREM1*) and four upregulated genes (*RGS4*, *ABCA12*, *CDH1*, and *CLDN1*), alongside *RHOA*, were selected for RT‒qPCR analysis. The results confirmed significant differential expression of these genes between groups (Figure 4D), with expression patterns notably aligning with those derived from the RNA-seq approach (Figure 4E).

**Table 2:** **Overview of the quality control and alignment results for the clean read data**

Sample	Total reads	Clean reads	Q20 (%)	Q30 (%)	GC (%)	Total mapped ratio (%)
WT-1	58 950 486	58 950 486	99.12	97.07	50.92	91.54
WT-2	64 434 188	64 434 188	99.16	97.19	50.06	91.93
WT-3	54 143 398	54 143 398	99.03	96.76	50.61	94.34
*RHOA*-KO-1	55 885 346	55 885 342	99.11	97.00	49.88	89.93
*RHOA*-KO-2	52 474 814	52 474 812	99.11	96.99	50.30	89.36
*RHOA*-KO-3	61 361 252	61 361 252	99.15	97.13	49.88	89.38

### Functional annotation of DE mRNAs

**Figure 5:**
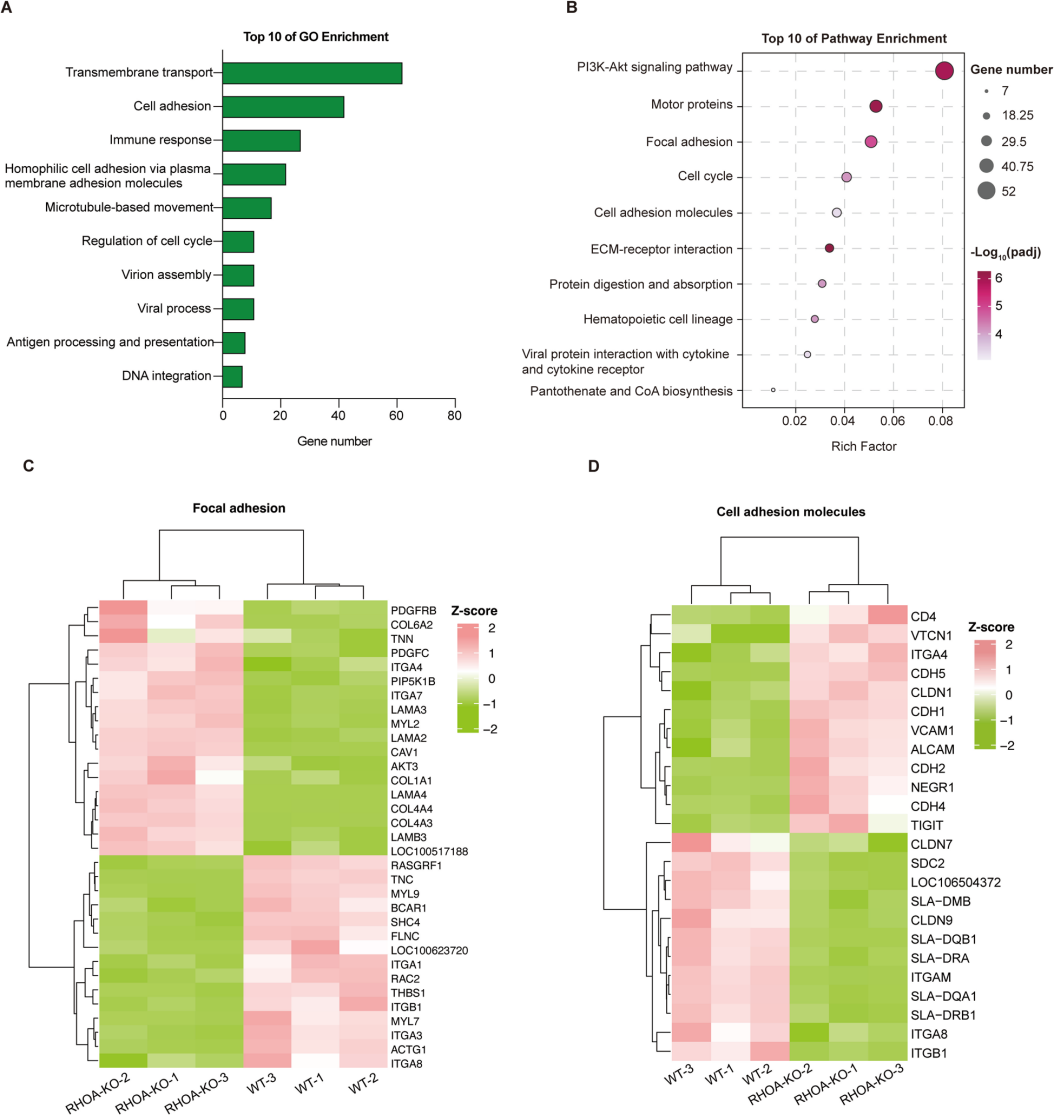
**Functional annotation and enrichment analysis of DE mRNAs. A** Top 10 significant GO terms for DE mRNAs. The y-axis displays GO biological processes, whereas the x-axis shows the enrichment factor. **B** Top 10 significantly enriched KEGG pathways for DE mRNAs. The y-axis lists the pathway names, and the x-axis indicates the enrichment factor. **C, D** Bi-clustering heatmap analysis illustrating the expression patterns of DE genes in the respective pathways.

Gene Ontology (GO) enrichment highlighted processes linked to cellular adhesion and cytoskeletal organization, including cell adhesion, homophilic cell adhesion via plasma membrane adhesion molecules, and microtubule-based movement processes (Figure 5A). The KEGG analysis revealed enrichment of cell adhesion molecules and focal adhesion signaling pathways (Figure 5B). The expression patterns of key genes in these pathways, including those encoding downregulated actin regulators (*MYL7*, *MYL9*, and *ACTG1*) and upregulated tight junction components (*CDH1*, *CLDN1*, and *CDH5*), were consistent with enhanced barrier function and impaired cytoskeletal remodeling (Figures 5C, D; Additional file 1).

## Discussion

*G. parasuis* causes systemic inflammatory responses that are severely detrimental to the health of pig populations. However, the specific pathogenic mechanisms remain unclear. In this study, we focused on *RHOA* as a host-dependent candidate gene and utilized LLC-PK1 cells as an in vitro infection model to explore the effects of *RHOA* knockout on *G. parasuis* infection and the underlying mechanisms.

Our findings reveal for the first time that the depletion of porcine *RHOA* effectively resists *G. parasuis* infection. mRNA transcriptome analysis revealed that *RHOA* knockout significantly enriched cell adhesion pathways, with genes related to tight junctions (TJs), including *CDH1* (encoding E-cadherin), *CLDN1* (encoding claudin-1), and *CDH5* (encoding VE-cadherin), being notably upregulated. These findings suggest that *RHOA* knockout may strengthen tight junction structures. In epithelial cells, TJs serve as a primary barrier against pathogens. However, many viruses have evolved mechanisms to exploit readily accessible structural proteins at the apical region to enter cells and facilitate their systemic spread. For example, the human hepatitis C virus uses tight junction proteins, including occluding [30] and claudin-1 [31], as entry factors into host cells. The West Nile virus disrupts intercellular connections by degrading various tight junction and adherens junction proteins, such as ZO-1, occludin, claudin-1, and VE-cadherin, allowing it to enter the bloodstream [32]. Similarly, *G. parasuis* disrupts occludin, ZO-1, E-cadherin, and VE-cadherin, damaging intercellular junctions and increasing permeability [1, 8, 33]. These findings indicate that *G. parasuis* compromises the ability of TJs to infect cells. The absence of *RHOA* may hinder the spread of *G. parasuis* between cells through TJs to effectively prevent invasion.

The disassembly of tight junctions is caused primarily by the contraction of the actomyosin ring, a dense circumferential band formed by F-actin and myosin II at the levels of TJs and adherens junctions [34]. The activation of the actomyosin ring is directly regulated by the RhoA/ROCK/MLC signaling pathway to provide the force necessary to disrupt TJs [2]. In this study, following *RHOA* knockout, the mRNA levels of *MLY7* and *MLY9*, which are involved in MLC synthesis, were decreased. Moreover, the mRNA levels of *ACTG1*, which encodes actin, significantly decreased. This could impede actin polymerization, thereby stabilizing TJs to fight *G. parasuis* infection. Many viruses, such as porcine sapovirus (PSaV), utilize the RhoA/ROCK/MLC signaling pathway to disassemble TJs and subsequently enter cells by binding to claudin-1 [35]. E-cadherin, located on the basolateral surface of polarized intestinal cells, serves as a receptor for the *Listeria* surface protein InlA [36]. Thus, the opening of TJs is necessary to briefly expose E-cadherin at the apical surface for bacterial internalization. Given that *G. parasuis* infection is associated with the disruption of TJs, we speculate that some receptor genes facilitating *G. parasuis* entry into host cells may be concealed beneath TJs.

Notably, TEM revealed *G. parasuis* invasion of porcine LLC-PK1 cells via pseudopod-like membrane protrusions traversing intercellular spaces, a mechanism conserved with bacterial pathogens including *Yersinia*, *Listeria*, *Shigella*, and *Salmonella*, which exploit *RHOA*-dependent processes involving actin cytoskeleton rearrangement and pseudopod formation for host cell entry [37–40]. In our study, *RHOA* knockout impaired this conserved process through: (1) significant enrichment of focal adhesion signaling pathways critical for cytoskeletal dynamics; (2) downregulation of actin-regulatory genes (*ACTG1*, *MYL7*, *MYL9*), reflecting *RHOA*'s positive regulation of actin polymerization via the ROCK/MLC pathway; and (3) consequent reduction in pseudopod formation and bacterial invasion, phenocopying *Shigella* suppression by Rho inhibitors [39, 40]. This pro-invasive function contrasts with *RHOA*’s pathogen-specific roles in porcine viruses: it promotes endocytosis and tight junction disruption during CSFV/PSaV infection [19–21] but inhibits PRV replication via cytoskeletal remodeling [22]. Collectively, *RHOA* serves as a universal invasion hub for bacteria through actin-remodeling mechanisms while exhibiting functional duality in viruses, highlighting RHOA/ROCK signaling as a prime target for intervention against Glässer’s disease. While our in vitro model delineates key mechanistic roles for *RHOA*, the essential protective function of *RHOA* depletion/inhibition and the therapeutic promise of targeting this pathway (e.g., via *ROCK* inhibitors or transcriptome-identified targets like *CDH1*/*ACTG1*) await comprehensive validation in in vivo porcine models.

In conclusion, the deletion of the host gene *RHOA* significantly reduces the adhesion and invasion of *G. parasuis* in host cells, thereby increasing cell survival. This mechanism may involve the regulation of the actin cytoskeleton and tight junctions through the RhoA/ROCK/MLC pathway. Our findings suggest that *RHOA* is an important candidate gene for the host response to *G. parasuis* infection. Further in vivo studies will be essential to confirm these observations and assess their translational relevance.

## Supplementary Information

**Additional file 1. KEGG pathway analysis of differentially expressed mRNAs related to focal adhesion and cell adhesion molecule signaling pathways. **The diagram illustrates key genes involved in the focal adhesion and cell adhesion molecule pathways, derived from KEGG enrichment analysis of RNA-seq data comparing RHOA-KO cells to WT controls.

**Additional file 2. List of DE mRNAs identified between the RHOA-KO and WT groups.**

## Data Availability

The datasets generated during the current study are available from the China National Center for Bioinformatic Repository (BioProject: PRJCA035746).
