# Supplementary figures and images for: A novel function of RHOA as a host-dependent factor in Glaesserella parasuis infection of LLC-PK1 cells
Source: Vet Res. 2025 Sep 25;56:179. doi: 10.1186/s13567-025-01617-6 (PMC12465878; doi:10.1186/s13567-025-01617-6)

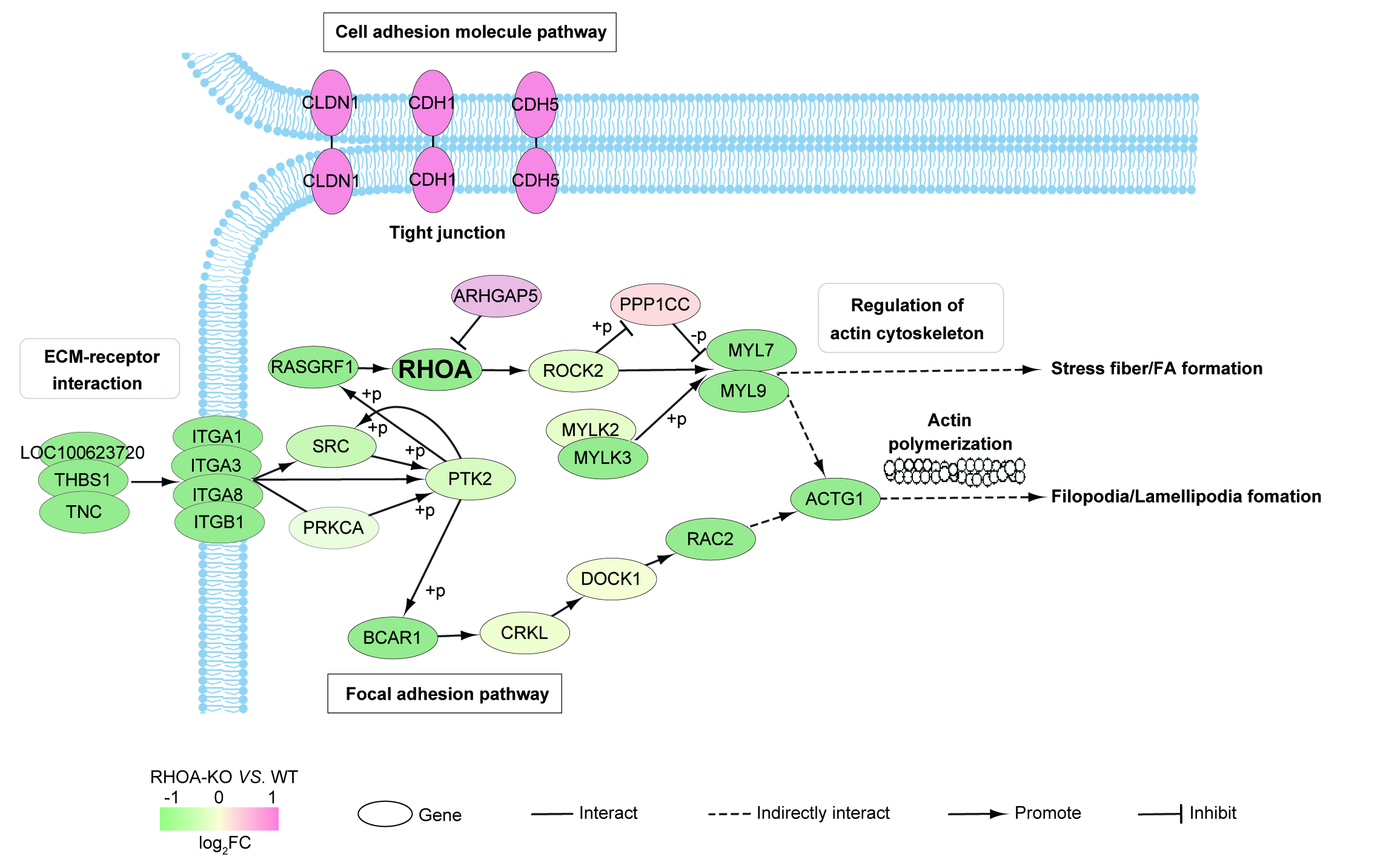

Supplement: Supplementary file 1 — Additional file 1. KEGG pathway analysis of differentially expressed mRNAs related to focal adhesion and cell adhesion molecule signaling pathways. The diagram illustrates key genes involved in the focal adhesion and cell adhesion molecule pathways, derived from KEGG enrichment analysis of RNA-seq data comparing RHOA-KO cells to WT controls. [file 13567_2025_1617_MOESM1_ESM.tif]
